# Hypogonadism and associated factors among male Leprosy patients

**DOI:** 10.1371/journal.pntd.0012374

**Published:** 2024-08-05

**Authors:** Nafyad Tolossa Urgie, Miftah Oumer Surur, Shimelis Nigussie, Beniam Worku Yigazu, Kidist Bobosha, Abebaye Aragaw, Getahun Shibru

**Affiliations:** 1 Department of Biomedical Sciences, College of Health Sciences, Arsi University, Asela, Ethiopia; 2 Alert Comprehensive Specialized Hospital, Addis Ababa, Ethiopia; 3 Armauer Hansen Research Institute, Addis Ababa, Ethiopia; 4 Department of Medical Physiology, School of Medicine, College of Health Sciences, Addis Ababa University, Addis Ababa, Ethiopia; Universidade Federal do Para, BRAZIL

## Abstract

**Background:**

Leprosy affects various organs in addition to skin, eyes, and peripheral nerves. Testicular involvement in leprosy patients is common and causes disturbance in endocrine function of the testis and results in hypogonadism. Hypogonadism is frequently undiagnosed and underreported.

**Objective:**

This study aimed to assess hypogonadism and associated factors among leprosy patients at Alert Comprehensive Specialized Hospital, Ethiopia.

**Methods:**

A cross-sectional study design was used in which consecutive 146 male leprosy patients aged between 18 to 65 years attending outpatient follow-up at leprosy outpatient clinic were included. Data was gathered both from patient charts and through patients’ interviews. Androgen deficiency symptoms were assessed by androgen deficiency in the aging male questionnaire, and 5ml of blood samples were taken from study participants and serum total testosterone, LH, and FSH were analyzed by Electrochemiluminescence method. Statistical correlation was assessed by Spearman correlation. A multivariable binary logistic regression model was used to identify the independent factors associated with hypogonadism and P-value <0.05 was used to declare statistical significance.

**Results:**

The prevalence of hypogonadism was 39 (26.7%). Out of this, 34 (87.2%) had primary hypogonadism, whereas 5 (12.8%) had secondary hypogonadism. Total testosterone was inversely correlated with Body mass index (r = -0.37, p = 0.002), Luteinizing hormone (r = -0.43, p <0.001), and Follicular stimulating hormone (r = -0.42, p< 0.001). However, Total testosterone was not significantly correlated with age (r = -0.019, p = 0.81). BMI [AOR = 1.32, 95%CI (1.16–1.51)] and grade-II disability [AOR = 3.80, 95%CI (1.23–11.64)] were identified as independent risk factors for hypogonadism.

**Conclusion:**

Nearly one-fourth of male leprosy patients had hypogonadism. Overweight and grade-II disability were independent risk factors for hypogonadism.

## Author summary

Leprosy affects various organs in addition to skin, eyes and peripheral nerves. Testicular involvement in leprosy patients is common and causes disturbance in endocrine function of the testis and results in hypogonadism.

Mycobacterium leprae reach the testicles through skin tissue invasion, blood or the lymphatic system, and the testes can serve as a reservoir for Mycobacterium leprae. A lower testicular temperature than internal body temperature may promote M. *leprae* growth in the testicle. It causes thickening and constriction of vessels, interstitial fibrosis, hyalinization and obliteration of seminiferous tubules and Leydig cells. These cause low testosterone productions among leprosy patients.

The prevalence and risk factors of hypogonadism among male leprosy patients vary in different settings. In this study area the prevalence of hypogonadism was 26.7%. Overweight and grade-II disability were identified as independent risk factors for hypogonadism.

## Introduction

Leprosy is a chronic infectious disease that primarily affects the skin, peripheral nerves, mucosal surfaces of the upper respiratory tract, and the eyes, and is known to affect people of all ages, from infancy to old age [1]. The incidence and prevalence of this disease vary greatly by nation, with developing countries bearing the burden of both new cases and patients on treatment [2]. According to the 2022 report by the World Health Organization (WHO), there were 174,087 newly reported cases of leprosy across 184 countries. Among these cases, 106,430 (61.0%) were male. The majority of countries experiencing high prevalence of new case detection are in the African and South-East Asia Regions [3]. Complications of leprosy include, sensory and motor nerve damage, blindness, nasal stiffness and septal perforation, renal disease, and testicular atrophy [4,5]. Because testes can operate as a reservoir for the lepra bacilli and immune-induced testicular cell death, leprosy can affect testicular endocrine function and cause hypogonadism [6]. Hypogonadism is a clinical and biochemical disorder characterized by symptoms and findings resulting from androgen deficiency caused by insufficient testosterone production due to different medical disorders, congenital or old age [7,8]. Hypogonadism has a significant negative impact on one’s quality of life by negatively influencing a variety of systems. The prevalence of hypogonadism ranges from 2.1% to 40% in middle-aged to older men, with an estimated incidence of 12 new cases per 1,000 person-years. Patients with concurrent illness have higher prevalence [9]. Because of the high prevalence of symptomatic hypogonadism, there is a significant public health burden in terms of sexual function and possible infertility [10]. The problem of hypogonadism is linked to not only sexual and reproductive function but also to depression, anemia, osteoporosis, fractures, frailty, metabolic syndrome and increased risk of cardiovascular mortality [11,12]. Male sexual drive and performance are significantly reduced when plasma testosterone levels fall below the normal range [13]. Even though hypogonadism has a detrimental health impact on leprosy patients, open discussion of sexually related concerns is frowned in Ethiopia. As a result, most patients are hesitant to discuss sexual issues with their doctor. As a result, hypogonadism is frequently silent, unreported, or undiagnosed and under-treated in these patients.

Although some studies have reported the prevalence and predictors of different complications of leprosy among leprosy patients. However, the involvement of sex hormones among leprosy patients is not widely studied and there is no study conducted to assess hypogonadism and associated factors among leprosy patients in Ethiopia. Therefore, this study aimed to assess hypogonadism and associated factors among leprosy patients.

## Methods

### Ethical considerations

Ethical clearance and approval were obtained from the Research and Ethical Review Committee of the Department of Physiology, School of Medicine, College of Health Sciences, Addis Ababa University and AHRI/Alert Ethics Review Committee (Ref No. PO-42-22). Written informed consent was obtained from each respondent after he was informed about the study. The information of the respondents was kept confidential.

### Study location

The study was conducted at Alert Comprehensive Specialized Hospital which was a WHO accredited international leprosy training and rehabilitation center in Ethiopia. It is located southwest of Addis Ababa at an altitude of 2,303 meters above sea level. It provides a variety of outpatient and inpatient services. Outpatient clinics give outpatient services for leprosy follow-up clinics and general medical clinics for leprosy patients and other medical and surgical diseases.

### Study design, population and sample size

Institution based cross-sectional study design was employed. 18–65 years old male leprosy patients who were on the treatment and finished treatment (released from treatment) were included. Patients on testosterone replacement therapy, with history of pelvic chemotherapy, radiation, and mechanical testicular damage, and patients who had been diagnosed with chronic illnesses such as DM, liver cirrhosis, cancer, and AIDS were excluded.

The sample size was calculated using Epi Info 7; using a single proportion formula by using a 16% prevalence of hypogonadism among leprosy patients in Bangladesh [14], 95% confidence interval (CI), margin of error(d) of 5% and 10% non-response was added to the total sample size is computed and the calculated sample size was 226.

The source population was less than 10,000(N = 420), and then the sample size was corrected by using the correction formula, the corrected sample size was 146.

All consecutive male leprosy patients attending follow-up at Alert Hospital from January 01, 2023 to March 30, 2023 were included until the required sample size of 146 was obtained.

### Data collection procedures

Data was collected from the patient’s chart and by interview. After getting written informed consent, participants were interviewed by using a structured questionnaire on sociodemographic, behavioral characteristics and symptoms of hypogonadism.

Symptoms of hypogonadism were assessed by using Androgen Deficiency in the Aging Male (ADAM) questionnaire. ADAM questionnaire is the most widely used androgen deficiency screening tool. It has ten questions that evaluate the low androgen symptoms. With low testosterone levels, it exhibits low variable specificity but high sensitivity. Participants responded yes to erectile dysfunction and loss of libido questions or yes to three of any Androgen Deficiency in Aging Male (ADAM) questionnaire considered as ADAM positive [15].

5ml of venous whole blood samples were taken from the participants early in the morning, before 11 AM and left for thirty minutes to clot and then Centrifuged at 1500 rpm to separate the serum. Separated serum was allocated for the chemistry test. Using the fully automated Cobas e411 analyzers, serum total testosterone (TT) luteinizing hormone (LH), and follicular stimulating hormone (FSH) were analyzed with Electrochemiluminescence (ECL) technology. TT≤12.1nmol/L was considered as low total testosterone [16]. Hypogonadism was defined as the presence of hypogonadism symptoms (ADAM positive) and low serum total testosterone (TT≤12.1nmol/L)[16]. Hypogonadal subjects were further classified. Hypogonadal subjects with elevated serum FSH (>14 mIU/ml), LH (>7.8 mIU/ml) or both as primary hypogonadism and Hypogonadal subjects with either low or normal FSH (≤14 mIU/ml), LH (≤7.8 mIU/ml) or both as secondary hypogonadism [16].

### Statistical analysis

The data was entered in EpiData version 4.6 and then exported to Stata version 14.0 for analysis. Means and standard deviations were used to present the descriptive statistics, in contrast percentages and frequencies were used to display categorical variables. After the normality test was done by Kolmogorov-Smirnov and Shapiro-Wilk test. All continuous variables failed to have a normal distribution even after being logarithmically transformed. Then a comparison of categorical independent variables between the hypogonadal group and eugonadal group was done by chi square test and comparison of continuous independent variables between the hypogonadal group and eugonadal group was done by mann whitney U test as well as statistical correlation between total testosterone(TT) and continuous independent variables was checked by Spearman correlation. Logistic regression analysis was employed to evaluate statistical relations. To determine the existence of crude association, the bivariate logistic regression analysis was applied. Variables that were clinically significant and had a P<0.25 in the bivariate logistic regression analysis were chosen to be included in the multivariable logistic regression. The independent variables contributed to hypogonadism were assessed using a multivariable logistic regression analysis. Both adjusted odd ratios and crude odd ratios with 95% CI were depicted as summary measures and Statistical significances were considered at a P-value < 0.05.

## Results

A total of 146 male leprosy patients were enrolled in this study. Participants’ age ranged from 20 to 65 years with the mean age of 41 ±13SD and 40 (27.4%) of study participants’ age ranged between 50–65 years. As summarized in Table 1, the majority of these participants 112 (76.7%) were married.

**Table 1:** Sociodemographic characteristics of participants (n = 146).

Variables	Frequency	Percentage
**Age Categories**		
20–34	54	37.0
35–49	52	35.6
50–65	40	27.4
**Marital status**		
Married	112	76.7
Single	29	19.9
Widowed and Divorced	5	3.4
**Level of education**		
No formal education	35	23.9
Primary	72	49.3
Secondary and above	39	26.8
**Occupation**		
Daily laborer	19	13.0
Others (beggar, no work, NGO employee)	22	15.1
Merchant	26	17.8
Farmer	38	26.0
Government employee	41	28.1
**Income per month(USD)**		
<27	35	24.0
27–87	98	67.1
Above 87	13	8.9

Behavioral and clinical profile of Study Participants summarized in the Table 2, the majority of respondents, 142 (97.3%), had multibacillary (MB) leprosy and 69 (47.3%) were diagnosed in the past five years. Out of the total study participants, 16 (10.9%) had a history of khat chewing. Out of those who had a history of khat chewing, 11 (7.5%) had chewed khat for more than 5 years and 5 (3.4%) had a history of cigarette smoking.

**Table 2:** Behavioral and clinical profile of Study Participants (n = 146).

Variables	Frequency	Percentage
**Leprosy type(WHO classification)**		
Paucibacillary (PB)	4	2.7%
Multibacillary (MB)	142	97.3%
**Duration since diagnosed leprosy**		
1month to 5years	69	47.3%
>5 years	77	52.7%
**Treatment of leprosy**		
On treatment (MDT)	46	31.5%
Completed MDT	100	68.5%
**Disability grade**		
Grade-0	28	19.2%
Grade I	61	41.8%
Grade II	57	39.0%
**Presence of leprosy reaction**		
Type I	28	19.2%
Type II	11	7.5%
History of diagnosed chronic non communicable disease	9	6.2%
**BMI(mean±SD)**		22kg/m^2^ ±3.3kg/m^2^
Underweight	7	4.8%
Normal	114	78.1%
Overweight	25	17.1%
History of taking other drugs (not including MDT and prednisolone).		
No	114	78.1%
Yes	32	21.9%
**Khat chewing history**		
Yes	16	11.0%
No	130	89.0%
**Cigarette Smoking history**		
Yes	5	3.4%
No	141	96.6%

MDT: Multidrug therapy; BMI: body mass index; SD: Standard deviation

Other drugs; (ibuprofen, omeprazole, hydrochlorothiazide, and nifedipine)

**Fig 1:**
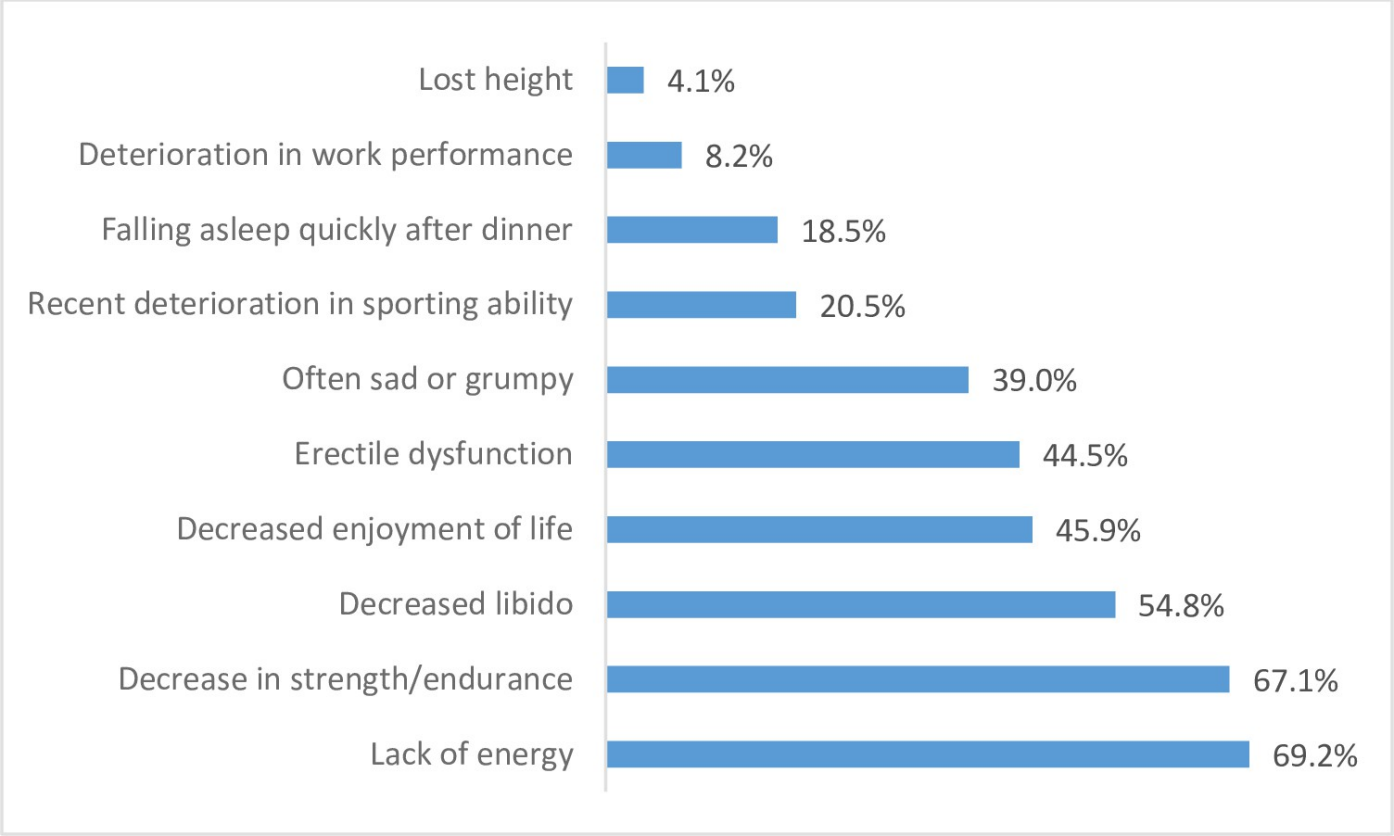
Androgen Deficiency Symptoms among Study Participants (n = 146).

The prevalence of androgen deficiency symptoms among the study subjects were summarized in Fig 1. The most reported symptom was lack of energy 101 (69.2%) followed by a decrease in strength/endurance 98 (67.1%). The two relatively more specific symptoms: loss of libido was reported by 80 (54.8%) of the study participants and erectile dysfunction was reported by 65 (44.5%) of study participants.

Hormonal profiles of study participants were summarized in Table 3; mean of total testosterone was 22.6 nmol/L ±11.6 nmol/L SD.

**Table 3:** Hormone data of study participants among male leprosy patients. (n = 146).

Hormones	Mean±SD	Minimum value	Maximum value
**TT (nmol/L)**	22.6±11.6	4.4	45.1
**LH (mIU/ml)**	7.76±5.05	1.62	33.6
**FSH (mIU/ml)**	12.7±5.65	4.98	36.2

TT: Total testosterone LH: Luteinizing hormone FSH: Follicular stimulating hormone

SD: Standard deviation

Based on the classification criteria for responses to the ADAM (Androgen deficiency in aging males) questionnaire described in the methodology section, 115 (78.8%) of study participants were ADAM positive, and the remaining 31 (21.2%) were ADAM negative.

Out of the total 146 study participants, only 39 (26.7%) had a low TT level (TT≤12.1nmol/L) and were positive for ADAM (Table 4). Only 3 (2.05%) of the ADAM negative participants had low TT level (Table 4). Therefore, only 26.7% (95%CI: 19.7%–34.7%) of study participants met the current definition of hypogonadism, which includes the presence of symptoms and a low testosterone level. Out of the participants who had hypogonadism, 34 (87.2%) had primary hypogonadism, whereas 5 (12.8%) had secondary hypogonadism.

**Table 4:** Testosterone Level Group in ADAM Positive and ADAM Negative Study Participants (n = 146).

ADAM Questionnaires’ response	Testosterone level	
	Low (TT≤12.1nmol)	Normal (TT>12.1nmol/L)
ADAM Positive (n = 115)	39 (26.7%)	76 (52.05%)
ADAM Negative (n = 31)	3 (2.05%)	28 (19.2%)

Within parentheses are percentages over grand total

ADAM: Androgen deficiency in aging male TT: Total testosterone

Comparison of variables between the eugonadal and hyogonadal participants indicated BMI was significantly higher in the hypogonadal group (median: 24.6kg/m^2^) than in eugonadal group (median of 20.4kg/m^2^), p = 0.002. LH levels were also significantly higher among the hypogonadal group (median: 10.3mIU/ml) than in the eugonadal group (median: 6.0mIU/ml) P<0.001. Similarly, FSH levels were significantly higher in the hypogonadal group (median: 19 mIU/ml) than in the eugonadal group (median: 10.0 mIU/ml). P<00.1. However, Age differences between the two groups were not statistically differ p = 0.725. Grade-II disability and history of leprosy reaction were significantly high among the hypogonadal group [hypogonadal vs eugonadal: grade II disability 64.1% vs. 29.9%, p = 0.001; leprosy reaction 41% vs. 23.4%, p = 0.036] (Table 5).

**Table 5:** Comparison of variables of the participants with hypogonadal and eugonadal group (n = 146).

Non-Parametric data	Hypogonadal group (n = 39)	Eugonadal group (n = 107)	P-value
Age (years, median and IQR)	41 (30–50)	38 (29–50)	0.725
BMI (kg/m2, median and IQR)	24.6 (20.2–27.7)	20.4 (19.5–22.3)	0.02
LH (mIU/ml, median and IQR)	10.3 (8.8–15.1)	6.0 (4.6–6.8)	<0.001
FSH (mIU/ml, median and IQR)	19.2 (15.7–22.7)	10.0 (8.6–12.0)	<0.001
>5 years after being diagnosed with leprosy	21 (53.8%)	55 (51.4%)	0.80
Released from treatment	29 (74.4%)	71 (66.4%)	0.85
Grade II disability	25 (64.1%)	32 (29.9%)	0.001
Having History of leprosy reaction	16 (41%)	25 (23.4%)	0.036
Taking other drugs (not including MDT and prednisolone)	11 (28.2%)	21 (19.6)	0.27

Within parentheses are percentages over column total IQR: Interquartile range

BMI: Body mass index; LH: luteinizing hormone; FSH: follicular stimulating hormone

MDT: Multidrug therapy

All continuous variables used in this investigation, including age, BMI, TT, LH, and FSH, were checked for normality distributions. All of these variables failed to have a normal distribution even after being logarithmically transformed. Then Spearman’s correlation analysis was employed to assess the correlation of TT and continuous independent variables including age, BMI, LH and FSH. TT was inversely correlated with BMI (r = -0.37, p = 0.002), LH (r = -0.43, p<0.001) and FSH (r = -0.42, p< 0.001). However, TT was not significantly correlated with age (r = -0.019, p = 0.81).

Those variables that showed significant association with hypogonadism in bivariate analysis at p<0.25 were again analyzed on multivariate logistic regression analysis. As summarized in Table 6, in multivariate logistic regression, disability grade and larger BMI were factors significantly associated with hypogonadism at p<0.05.

**Table 6:** Multivariate Logistic Regression Analysis for Selected Factors Associated with Hypogonadism in Bivariate Logistic Regression among study participants (n = 146).

Variables	Categories	Hypogonadism		COR (95%CI)	AOR (95%CI)	p-value
		Yes	No			
Disability grade	Grade-0	5	23	1	1	
	Grade-I	9	52	0.80 (0.24–2.64)	0.89 (0.26–3.02)	0.85
	Grade-II	25	32	3.59 (1.2–10.79)	3.8 (1.23–11.64)	0.02*
History of leprosy reaction	No	23	82	1	1	
	Yes	16	25	2.28 (1.05–4.98)	2.24 (0.98–5.12)	0.06
History of taking other drugs	No	28	86	1	1	
	Yes	11	21	1.61 (0.69–3.75)	1.46 (0.59–3.57)	0.41
BMI (mean±SD)		24±4.1	21±2.5	1.33 (1.17–1.50)	1.32 (1.15–1.51)	0.001*

1 = indicate for reference group * = indicate for significance at p<0.05

BMI: Body mass index

SD: standard deviation

## Discussion

This study assessed hypogonadism among male leprosy patients using the recent definition of hypogonadism and testosterone cut-off value of International Society of the Aging men. Our findings are alarming, with nearly one-fourth of male leprosy patients had hypogonadism and primary hypogonadism occurred in approximately four fifth whereas, hypogonadotropic hypogonadism occurred in one-fifth of them. BMI, LH and FSH were negatively correlated with total testosterone and this study demonstrated that grade-II disability and overweight were independent risk factors for hypogonadism.

The prevalence of hypogonadism among male leprosy patients was 26.7% (n = 146, 95%CI: 19.7%– 34.7%), which is higher than in the study done in Bangladesh 16.2% [14]. Several factors could be the reason for the higher prevalence and difference in the aforementioned study. The first contributor might be the small sample size in a study conducted in Bangladesh compared to our study. Another reason could be differences in selected study participants, in the study conducted in Bangladesh, the time of onset of symptoms for most of the participants was less than five years when compared to our study: because, the higher prevalence of hypogonadism in male leprosy patients has been confirmed to be significantly contributed by chronic leprosy cases [17].

In contrast, the prevalence of hypogonadism in this study was lower than in studies conducted in ‘Brazil 37.5% [18], India 39.5% [19], Indonesia 40.6% [20], and Turkey 51% [8], this discrepancy might be due to many factors-: All of these studies didn’t use the current definition of hypogonadism stated by International Society For the Study of Aging Male (ISSAM), which includes sex hormone level and symptoms of hypogonadism together, and in some studies, the cut-off point for low testosterone was not stated. One factor contributing to the difference in prevalence between this study and previous studies could be the age of the study participants. In a study conducted in Brazil, 47% of study participants’ age ranged from 60 to 75 years (mean 48.43±18.65 SD), and in a study conducted in Turkey, (mean age: 58±10.5 years, which is higher compared to this study (mean age: 40±13.05SD). Advanced age was a contributing factor in the decrease of the testosterone level [21].

Another reason for the higher prevalence in previous studies could be the cut-off point for low total testosterone. In a study conducted in India 39.5% [19], the cut-off point for low testosterone was 6ng/ml, which is higher than the cut-off point used in this study. The reason for the higher prevalence of hypogonadism in the study conducted in Turkey could be the duration of disease of the participants included in the study. Only male leprosy patients diagnosed 5 years before the time of the study were included, and the average duration of the disease was 36±11.68 years because, chronic leprosy cases were important contributors to the higher prevalence of hypogonadism in leprosy patients [17].

The prevalence of hypogonadism obtained in this study was nearly in line with the prevalence of hypogonadism obtained from studies conducted in India, 25.8% [17], and Bangladesh, 30.0% [22].

In this study, 5 patients had hypogonadotropic hypogonadism, 2 of whom were 60 years old and 3 of whom were above 50 years old. The observed hypogonadotropic hypogonadism could have been attributed to age rather than direct bacillary involvement. However, there were few studies that found low testosterone with low FSH and/or LH [23], and low testosterone with normal FSH and/or LH among leprosy patients [20]. This shows the need for advanced research on hypogonadotropic hypogonadism and leprosy.

In this study, age, time of diagnosis, type of leprosy, MDT status, and history of taking other drugs (not including MDT and prednisolone) did not differ significantly between the hypogonadal and eugonadal groups. However, grade-II disability, history of leprosy reaction, BMI, LH and FSH were significantly higher among hypogonadal groups compared to eugonadal groups. A similar study in Bangladesh partly agrees with our finding that grade-II disability, LH, and FSH were significantly high among hypo gonadal groups but not with history of leprosy reaction [14]. Another study conducted in Bangladesh was also partly in line with our finding history of leprosy reaction (ENL), LH and FSH were significantly higher among hypogonadal group compared to eugonadal group but frequency disability grade was not differ significantly among groups [22]. Many studies conducted in this area did not consider BMI as an influencing variable of hypogonadism among leprosy patients. In our study, BMI was significantly high among hypogonadal group. Because high BMI was confirmed to be an important factor of hypogonadism [24].

In this study, it is found that BMI, LH and FSH were negatively correlated with total testosterone. This finding was partly in apparent agreement with studies in India [19], Bangladesh [14], Indonesia [20]. Vascular thickening and fibrosis of testicular tissue could due to M. leprae testicular infiltration and change in the immune response driven by inflammatory cytokines, as well as local alterations cause significant negative correlation between TT and LH, and TT and FSH. This has an impact on the Leydig cells as well as the seminiferous tubules [25]. An increase in LH and a decrease in testosterone have a definite causal connection, since testosterone controls the release of LH through negative feedback, so between testosterone and LH, there was a significant negative connection. FSH and testosterone also correlated negatively, despite the fact that testosterone does not control FSH secretion, this could indicate that Sertoli cell destruction and Leydig cell damage happen simultaneously [26].

This study found that grade-II disability and BMI remained significantly associated risk factors of hypogonadism. Excess adipose tissue in overweight and obesity causes an increase in aromatase enzyme activity, which converts testosterone to estradiol (E2). Estrogens reduce the amount of testosterone produced overall by inhibiting the release of GnRH from the hypothalamus, as well as LH and FSH from the pituitary, through a negative feedback mechanism [27].

The secretion of leptin and pro-inflammatory cytokines is also increased with increased visceral fat. Pro-inflammatory cytokines cause Leydig cell destruction, directly impair LH function, and decrease GnRH release from the hypothalamus, which decreases testosterone levels. Leptin has receptors in the hypothalamus and Leydig cells that suppress the secretion of GnRH from the hypothalamus and testosterone secretion in Leydig cells [28]. These could be clear evidence for the reason why this study identified an increase in BMI as a risk factor for hypogonadism.

Even though it did not reach a level of statistical significance, history of reaction (p = 0.06) was also observed to be in higher frequency among those with hypogonadism in this study. A possible explanation for this could be that participants on MDT for leprosy were included in this study. Because reactions can happen both before and after MDT but more significant after completion of MDT [29], the importance of reaction as risk factor of hypogonadism may be reduced by their inclusion. An increased disability grade was associated with a higher bacillary load and leprosy reaction [30]. When there is a higher bacillary load, bacillary testicular infection increases since M. *leprae* prefers low temperatures in testicles and causes testicular tissue damage. Leprosy reaction was also associated with grade-II disability that could contribute to testicular damage through deposition of the immune complex in testicles or direct attack of the testes by pro-inflammatory cytokines [26]. This could be possible evidence for the reason why this study identified a grade-II disability as risk factor for hypogonadism. Thus, clinical conditions like grade-II disability and overweight may help in the early detection of hypogonadism in male leprosy patients.

This study is limited by the failure to include matched control group due to financial constraints, the inability to measure testicular volume due to lack of orchid meter, and the inability to perform semen analysis because of their religious belief. However, publications on hypogonadism in male leprosy patients are scarce; these findings could be very useful for future advanced studies.

In conclusion, nearly one-fourth of male leprosy patients had hypogonadism. BMI, LH and FSH were negatively correlated with total testosterone. Overweight and grade-II disability were independent risk factors for hypogonadism. Therefore, measurement of serum testosterone levels as part of the routine work-up in male leprosy patients should be recommended.

## Supporting information

S1 Fig The Frequency of Androgen Deficiency Symptoms Study Participants. (XLSX)

S1 Data Raw data of study participants. (XLSX)
